# Meal sequence and glucose excursion, gastric emptying and incretin secretion in type 2 diabetes: a randomised, controlled crossover, exploratory trial

**DOI:** 10.1007/s00125-015-3841-z

**Published:** 2015-12-24

**Authors:** Hitoshi Kuwata, Masahiro Iwasaki, Shinobu Shimizu, Kohtaro Minami, Haruyo Maeda, Susumu Seino, Koji Nakada, Chihiro Nosaka, Kenta Murotani, Takeshi Kurose, Yutaka Seino, Daisuke Yabe

**Affiliations:** Yutaka Seino Distinguished Center for Diabetes Research, Kansai Electric Power Medical Research Institute, 1-5-6 Minatojimaminamimachi, Chuo-ku, Kobe, 650-0047 Japan; Center for Diabetes, Endocrinology and Metabolism, Kansai Electric Power Hospital, Osaka, Japan; Center for Metabolism and Clinical Nutrition, Kansai Electric Power Hospital, Osaka, Japan; Division of Molecular and Metabolic Medicine, Department of Physiology and Cell Biology, Kobe University Graduate School of Medicine, Kobe, Japan; Department of Clinical Laboratory, Kansai Electric Power Hospital, Osaka, Japan; Department of Surgery, Jikei University School of Medicine, Tokyo, Japan; Kyowa Hakko Kirin Co. Ltd, Tokyo, Japan; Division of Biostatistics, Clinical Research Center, Aichi Medical University, Nagakute, Aichi Japan

**Keywords:** Gastric emptying, GIP, GLP-1, Meal sequence, Postprandial glucose variability

## Abstract

**Aims/hypothesis:**

Investigation of dietary therapy for diabetes has focused on meal size and composition; examination of the effects of meal sequence on postprandial glucose management is limited. The effects of fish or meat before rice on postprandial glucose excursion, gastric emptying and incretin secretions were investigated.

**Methods:**

The experiment was a single centre, randomised controlled crossover, exploratory trial conducted in an outpatient ward of a private hospital in Osaka, Japan. Patients with type 2 diabetes (*n* = 12) and healthy volunteers (*n* = 10), with age 30–75 years, HbA_1c_ 9.0% (75 mmol/mol) or less, and BMI 35 kg/m^2^ or less, were randomised evenly to two groups by use of stratified randomisation, and subjected to meal sequence tests on three separate mornings; days 1 and 2, rice before fish (RF) or fish before rice (FR) in a crossover fashion; and day 3, meat before rice (MR). Pre- and postprandial levels of glucose, insulin, C-peptide and glucagon as well as glucagon-like peptide-1 (GLP-1) and glucose-dependent insulinotropic polypeptide were evaluated. Gastric emptying rate was determined by ^13^C-acetate breath test involving measurement of ^13^CO_2_ in breath samples collected before and after ingestion of rice steamed with ^13^C-labelled sodium acetate. Participants, people doing measurements or examinations, and people assessing the outcomes were not blinded to group assignment.

**Results:**

FR and MR in comparison with RF ameliorated postprandial glucose excursion (AUC_−15–240 min_-glucose: type 2 diabetes, FR 2,326.6 ± 114.7 mmol/l × min, MR 2,257.0 ± 82.3 mmol/l × min, RF 2,475.6 ± 87.2 mmol/l × min [*p* < 0.05 for FR vs RF and MR vs RF]; healthy, FR 1,419.8 ± 72.3 mmol/l × min, MR 1,389.7 ± 69.4 mmol/l × min, RF 1,483.9 ± 72.8 mmol/l × min) and glucose variability (SD_−15–240 min_-glucose: type 2 diabetes, FR 1.94 ± 0.22 mmol/l, MR 1.68 ± 0.18 mmol/l, RF 2.77 ± 0.24 mmol/l [*p* < 0.05 for FR vs RF and MR vs RF]; healthy, FR 0.95 ± 0.21 mmol/l, MR 0.83 ± 0.16 mmol/l, RF 1.18 ± 0.27 mmol/l). FR and MR also enhanced GLP-1 secretion, MR more strongly than FR or RF (AUC_−15–240 min_-GLP-1: type 2 diabetes, FR 7,123.4 ± 376.3 pmol/l × min, MR 7,743.6 ± 801.4 pmol/l × min, RF 6,189.9 ± 581.3 pmol/l × min [*p* < 0.05 for FR vs RF and MR vs RF]; healthy, FR 3,977.3 ± 324.6 pmol/l × min, MR 4,897.7 ± 330.7 pmol/l × min, RF 3,747.5 ± 572.6 pmol/l × min [*p* < 0.05 for MR vs RF and MR vs FR]). FR and MR delayed gastric emptying (Time_50%_: type 2 diabetes, FR 83.2 ± 7.2 min, MR 82.3 ± 6.4 min, RF 29.8 ± 3.9 min [*p* < 0.05 for FR vs RF and MR vs RF]; healthy, FR 66.3 ± 5.5 min, MR 74.4 ± 7.6 min, RF 32.4 ± 4.5 min [*p* < 0.05 for FR vs RF and MR vs RF]), which is associated with amelioration of postprandial glucose excursion (AUC_−15–120 min_-glucose: type 2 diabetes, *r* = −0.746, *p* < 0.05; healthy, *r* = −0.433, *p* < 0.05) and glucose variability (SD_−15–240 min_-glucose: type 2 diabetes, *r* = −0.578, *p* < 0.05; healthy, *r* = −0.526, *p* < 0.05), as well as with increasing GLP-1 (AUC_−15–120 min_-GLP-1: type 2 diabetes, *r* = 0.437, *p* < 0.05; healthy, *r* = 0.300, *p* = 0.107) and glucagon (AUC_−15–120 min_-glucagon: type 2 diabetes, *r* = 0.399, *p* < 0.05; healthy, *r* = 0.471, *p* < 0.05). The measured outcomes were comparable between the two randomised groups.

**Conclusions/interpretation:**

Meal sequence can play a role in postprandial glucose control through both delayed gastric emptying and enhanced incretin secretion. Our findings provide clues for medical nutrition therapy to better prevent and manage type 2 diabetes.

**Trial registration:**

UMIN Clinical Trials Registry UMIN000017434.

**Funding:**

Japan Society for Promotion of Science, Japan Association for Diabetes Education and Care, and Japan Vascular Disease Research Foundation.

**Electronic supplementary material:**

The online version of this article (doi:10.1007/s00125-015-3841-z) contains peer-reviewed but unedited supplementary material, which is available to authorised users.

## Introduction

Postprandial glucose homeostasis is controlled by numerous factors including meal size and composition, gastric emptying and intestinal glucose absorption through the secretion and action of insulin and glucagon, and the incretins. As postprandial glucose elevation and variability are implicated in the pathogenesis of micro- and macro-vascular complications related to diabetes [1–3], they represent an important area of study from both a pharmacological and medical nutrition therapy perspective. The quantity and type of carbohydrate in a meal influences postprandial glucose excursion; the total amount of carbohydrate is the primary predictor of the glycaemic response [4]. However, recent studies indicate that preloading of foods such as vegetables, whey proteins and olive oil before carbohydrate intake can improve postprandial glucose excursions in type 2 diabetes [5–7], suggesting meal sequence as a novel target in management of postprandial glucose excursion in type 2 diabetes.

The gastric emptying rate is known to be critical in early and overall postprandial glucose excursions in individuals both with and without type 2 diabetes; even modest changes in the rate of gastric emptying or glucose entry into the duodenum have a substantial impact on glucose excursion after ingestion of various carbohydrates [8, 9]. Gastric emptying is known to be controlled by neuro-endocrine systems involving gut-derived hormones such as glucagon-like peptide-1 (GLP-1), cholecystokinin and peptide tyrosine tyrosine, which delay gastric emptying, and motilin and ghrelin, which accelerate gastric emptying [8, 9]. These gut-derived hormones mediate gastric emptying rate through macronutrient composition (fat, protein and carbohydrate) [8, 9]. Previous studies found that preload of whey protein or olive oil enhanced GLP-1 secretion and delayed gastric emptying in individuals both with and without type 2 diabetes [6, 7], suggesting that preload of small amounts of proteins or fats before meals might be effective in postprandial glucose control for diabetes. However, this model needs to be tested with more generally available, ordinary foods for such nutrition therapy to be practicable in patients’ daily lives.

Incretin is an important area of research for postprandial glucose management; the hormones are known to be responsible for more that 50–70% of insulin secretion after oral ingestion of glucose [10–12]. Glucose-dependent insulinotropic polypeptide (GIP) and GLP-1 are secreted from the gut in response to ingestion of nutrients including carbohydrates, proteins and fats, and enhance insulin secretion glucose-dependently to exert their glucose-lowering effects [10–12]. GLP-1 also delays gastric emptying rate and suppresses glucagon secretion, which ameliorates postprandial glucose elevation [10–12]. It has been demonstrated that GIP has no such effects, but facilitates fat accumulation, increasingly impairing glycaemic control [13]. Pharmacological interventions targeting incretins, such as dipeptidyl peptidase-4 inhibitors (DPP-4i) and GLP-1 receptor agonists (GLP-1RA), are widely used in the management of type 2 diabetes [11, 14]. However, nutritional intervention based on the incretins has not been established. Preload of some proteins or fats ameliorates postprandial glucose elevation through enhancement of GLP-1 secretion and delayed gastric emptying [6, 7, 15]. Previous studies in cultured cells and experimental animals also indicate that fish oils, eicosapentaenoic acid (EPA) and docosahexanoic acid (DHA), enhance GLP-1 secretion [16, 17]; we hypothesised that nutrients in fish might stimulate GLP-1 secretion and improve postprandial glucose elevation.

In this study, we compared the effects of fish before and after rice intake, in a crossover fashion, on postprandial glucose excursion, gastric emptying and incretin secretion in type 2 diabetes patients and healthy controls. We also included a meat before rice (MR) intake arm, as a comparator for the fish before rice (FR) arm, using meat with total calorie and protein/fat ratio similar to fish in the same individuals.

## Methods

The protocol (UMIN registration number: UMIN000017434) was approved by the ethics committee of Kansai Electric Power Hospital and written informed consent was obtained from all participants. The study was conducted according to the principles expressed in the Declaration of Helsinki.

### Participants

**Fig. 1:**
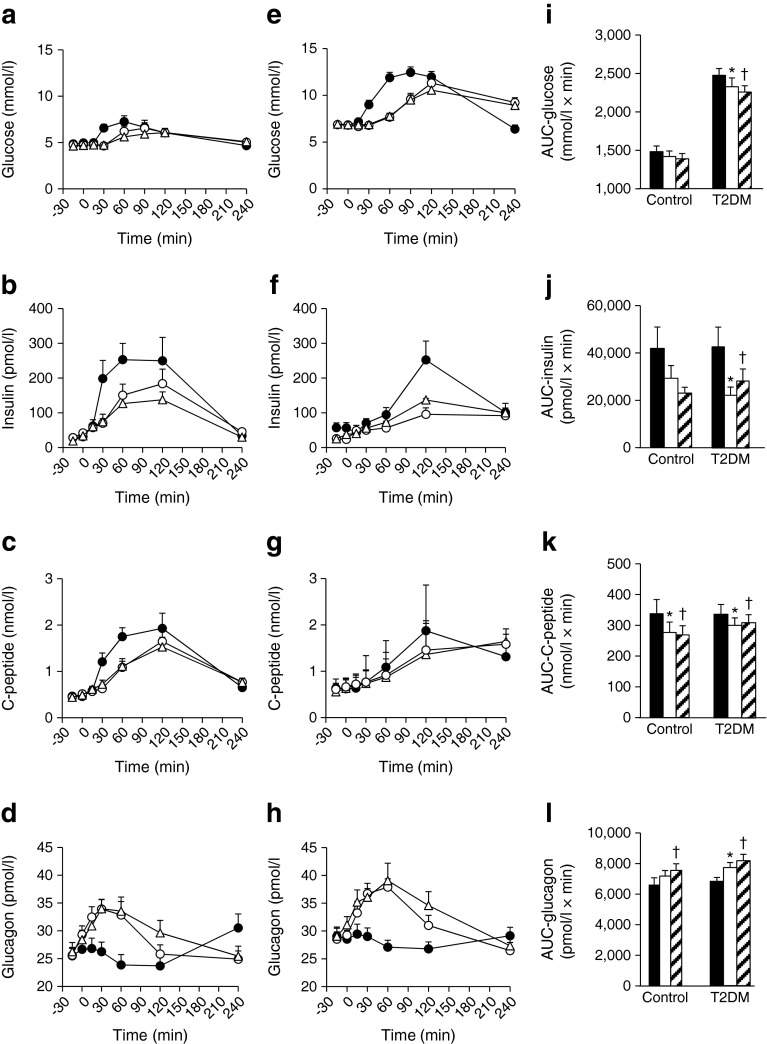
Time course curves are indicated for glucose, insulin, C-peptide and glucagon in healthy controls (**a**–**d**) and patients with type 2 diabetes (**e**–**h**) during three different meal sequences: RF, black circles; FR, white circles; or MR, white triangles (**a**–**h**). The *p* values for differences due to sequence (X), time (Y), and the interaction of sequence and time (Z), were calculated by mixed effects models as follows: (**a**) X0.000, Y0.001 and Z0.007; (**b**) X0.000, Y0.000 and Z0.012; (**c**) X0.000, Y0.000 and Z0.001; (**d**) X0.000, Y0.000 and Z0.000; (**e**) X0.000, Y0.000 and Z0.000; (**f**) X0.000, Y0.004 and Z0.614; (**g**) X0.000, Y0.163 and Z0.000; (**h**) X0.000, Y0.000 and Z0.000. AUC_−15–240 min_ are indicated (RF, black bars; FR, white bars; MR, hatched bars) (**i**–**l**). AUCs were analysed by Wilcoxon’s rank sum test; * and ^†^ indicate *p* < 0.05 for RF vs FR, and RF vs MR, respectively

**Fig. 2:**
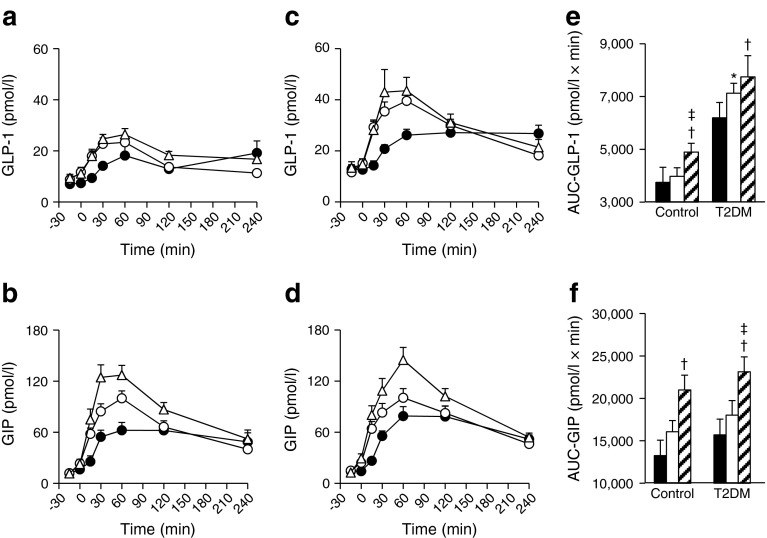
Time course curves are indicated for GLP-1 and GIP in healthy controls (**a**, **b**) and patients with type 2 diabetes (**c**, **d**) during three different meal sequences: RF, black circles; FR, white circles; or MR, white triangles (**a**–**d**). The *p* values for differences due to sequence (X), time (Y), and the interaction of sequence and time (Z), were calculated by mixed effects models as follows: (**a**) X0.000, Y0.000 and Z0.000; (**b**) X0.000, Y0.000 and Z0.000; (**c**) X0.000, Y0.000 and Z0.000; and (**d**) X0.000, Y0.000 and Z0.000. AUC_−15–240 min_ are indicated (RF, black bars; FR, white bars; MR, hatched bars) (**e**, **f**). AUCs were analysed by Wilcoxon’s rank sum test; *, ^†^ and ^‡^ indicate *p* < 0.05 for RF vs FR; RF vs MR; and FR vs MR, respectively

**Fig. 3:**
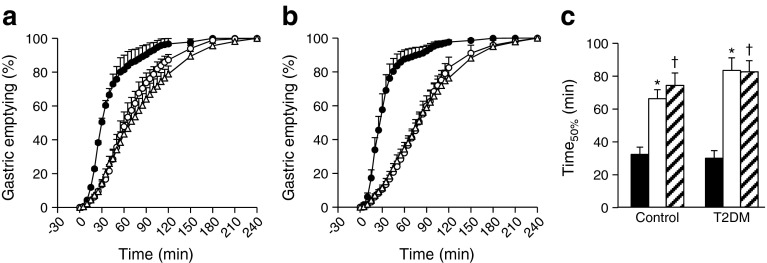
Time course curves are indicated for gastric emptying in healthy controls (**a**) and patients with type 2 diabetes (**b**) during three different meal sequences: RF, black circles; FR, white circles; or MR, white triangles (**a**, **b**). The *p* values for differences due to sequence (X), time (Y), and the interaction of sequence and time (Z), were calculated by mixed effects models as follows: (**a**) X0.000, Y0.000 and Z0.000; and (**b**) X0.000, Y0.000 and Z0.000. Half times for emptying the ingested steamed rice (Time_50%_) are indicated (RF, black bars; FR, white bars; MR, hatched bars) (**c**). Time_50%_ was analysed by Wilcoxon’s rank sum test; * and ^†^ indicate *p* < 0.05 for RF vs FR, and RF vs MR, respectively

**Fig. 4:**
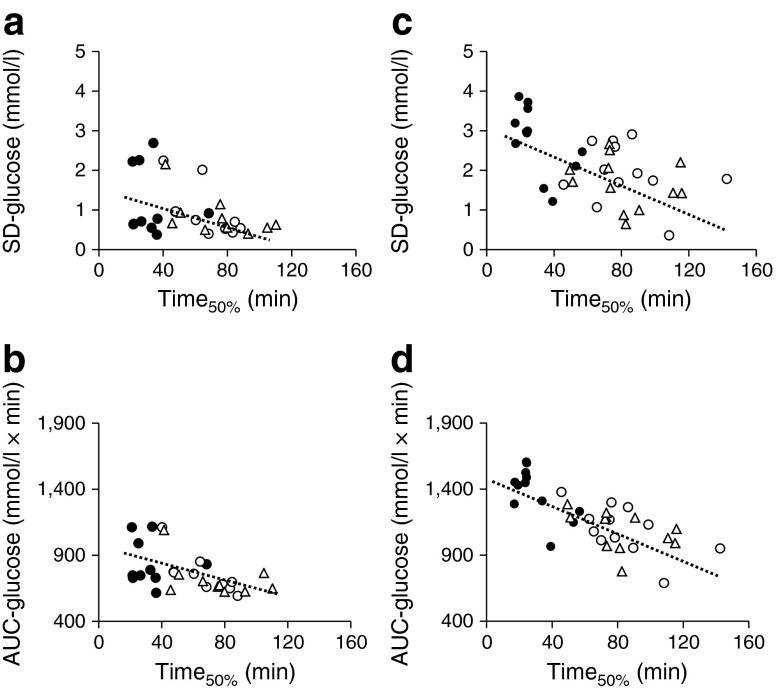
Scatter plots of SD_−15–240 min_-glucose (SD-glucose) and AUC_−15–120 min_-glucose (AUC-glucose) with time 50% during meal sequence tests (Time_50%_) were plotted (RF, black circles; FR, white circles; MR, white triangles) for healthy controls (**a**, **b**) and type 2 diabetes patients (**c**, **d**). The Pearson correlation coefficient (*r*) and *p* values were calculated, respectively, as follows: (**a**) *r* = −0.526, *p* < 0.05; (**b**) *r* = −0.433, *p* < 0.05; (**c**) *r* = −0.578, *p* < 0.05; and (**d**) *r* = −0.746, *p* < 0.05

Individuals with untreated type 2 diabetes and healthy volunteers aged 30–75 years, HbA_1c_ 9.0% (75 mmol/mol) or less, and BMI 35 kg/m^2^ or less were recruited to investigate the effects of meal sequence on postprandial glucose excursion, secretion of insulin, glucagon and the incretins and on gastric emptying (Table 1). At screening, participants with type 1 diabetes, gastrointestinal tract disease including gastroparesis, history of gastrointestinal operation, cardiac disease, pulmonary disease, pancreatic disease, liver disease, renal disease, alcohol or drug abuse, glucose-lowering medication, diabetogenic medication or malignancy, or pregnancy were excluded. Individuals allergic to mackerel were excluded. No participants had thyroid diseases. Diagnosis of type 2 diabetes accords to the criteria of the Japanese Diabetes Society [18]. No individuals demonstrated positive Schellong test or decreased coefficient of RR interval in electrocardiogram variation, suggesting the absence of diabetic autonomic neuropathy. Previous studies employing six to eight individuals with type 2 diabetes demonstrated that preload of whey protein or olive oil ameliorated postprandial glucose excursion, enhanced GLP-1 secretion and delayed gastric emptying [6, 7]. Our preliminary experiments employing 12–13 individuals with type 2 diabetes demonstrated that preload of fish or meat suppressed postprandial glucose elevation and enhanced GLP-1 secretion (electronic supplementary material [ESM] Figs 1-6). These results supported the current exploratory study sample size of 10–12 individuals each for controls and type 2 diabetes.

**Table 1:** Characteristics of individuals with type 2 diabetes and healthy controls participating in the current study

	Healthy controls	Type 2 diabetes
*n* (male/female)	10 (10/0)	12 (9/3)
Age (years)	38.4 ± 4.9	59.7 ± 9.7
BMI (kg/m^2^)	22.7 ± 2.4	25.3 ± 4.1
Duration of diabetes (years)	–	3.6 ± 5.5
Fasting plasma glucose (mmol/l)	4.86 ± 0.13	6.84 ± 0.31
HbA_1c_ (%) (mmol/mol)	5.4 ± 0.3 (35.1 ± 8.2)	6.6 ± 0.5 (49.1 ± 5.2)
HOMA-IR	1.1 ± 0.5	2.7 ± 2.2
HOMA-β	81.1 ± 27.7	55.1 ± 47.9
Systolic BP (mmHg)	115.3 ± 8.2	123.6 ± 11.4
Diastolic BP (mmHg)	72.4 ± 7.0	76.7 ± 10.5

Each value indicates the mean ± SD

### Meal sequence test

Participants were subjected to meal sequence tests in the morning after overnight fast on three separate days: FR and rice before fish (RF) on the first 2 days in a crossover manner, and MR on the third day. In FR and MR, participants first ingested 920 kJ of boiled mackerel or grilled beef (ESM Table 1) and, 15 min later, 1,004 kJ of steamed rice. In RF, participants first ingested 1,004 kJ of steamed rice followed by 920 kJ of boiled mackerel 15 min later. The time patients received steamed rice is defined as 0 (in the experiments described in ESM Figs 1-6, the time at which patients received the first dish was defined as 0). The boiled mackerel and grilled beef had similar calorie and protein/fat ratio with limited carbohydrates. The amounts of boiled mackerel, grilled beef and steamed rice were prepared such that total energy and nutrient balance (protein:fat:carbohydrate ratio) were within dietary recommendations by the Japan Diabetes Society [19]. To measure levels of glucose and selected hormones, blood samples were withdrawn at −15, 0, 15, 30 60, 90, 120 and 240 min and stored as described previously [20]. To analyse gastric emptying rate, end-tidal breath samples were collected into small exhalation bags (PYLORI-BAG20, Otsuka Electronics Company, Osaka, Japan) at −15 min, 0 min and every 5 min until 120 min, and every 30 min until 240 min as recommended by the Japan Society of Smooth Muscle Research [21]. End-tidal breath samples were also collected into three large exhalation bags (PYLORI-BAG1.3L, Otsuka Electronics Company) at −15 min to use as reference for exhalation gas analysis. The canned boiled mackerel (4901901145899, Maruha Nichiro Corporation, Tokyo, Japan) was obtained commercially and stored at room temperature until use. The grilled beef was prepared by grilling sirloins of beef on an iron plate and subdividing them into small pieces stored in vacuum packs at −20°C. The boiled mackerel and grilled beef were prepared without seasonings that might affect digestion or absorption of ingested foods. The rice was prepared by steaming with ^13^C-labelled sodium acetate (CLM-156-0, Cambridge Isotope, MA, USA) at a ratio of 150 g steamed rice and 200 mg of ^13^C-labelled sodium acetate, and was stored at −20°C until use. The boiled mackerel, grilled beef and steamed rice were microwaved before being served.

### Laboratory determinations

Hormones were measured using the following assays as described previously [20, 22]. Total GLP-1, human total GLP-1 (ver. 2) assay kit (K150JVC-2; Mesoscale Discovery, Gaithersburg, MD, USA); total GIP, human GIP (total) ELISA (EZHGIP-54K; Merck Millipore, Darmstadt, Germany); glucagon, glucagon RIA (GL-3K; Merck Millipore); insulin, lumipulse presto insulin (Fujirebio, Tokyo, Japan); and C-peptide, lumipulse presto C-peptide (Fujirebio). Gastric emptying rate was determined by mathematical modelling, based on changes of the ^13^CO_2_/^12^CO_2_ ratio in breath samples measured by an infrared spectral analyser (POCone, Otsuka Electronics Company) [23]. Stable ^13^C-labelled sodium acetate is emptied from the stomach following the trituration and liquefaction of the steamed rice, and is transported to the liver via the portal vein, where it is oxidised to ^13^CO_2_ and exhaled into breath. The Wagner–Nelson method was applied to adjust the time for ^13^CO_2_ distribution [8, 24]. Other laboratory measurements including HbA_1c_ and plasma glucose were done by standard assays.

### Calculations and statistical analyses

Results are reported as mean ± SE of the mean unless otherwise stated. AUC of each measurement was calculated according to the trapezoidal rule. Standard deviation (SD)-glucose was expressed as SD of glucose levels from −15 min to 240 min. All statistical calculations were performed using IBM SPSS for Windows ver. 22 (SAS Institute, Berkeley, CA, USA). Repeated measures were analysed by mixed effects model. AUCs, SDs, and half time for emptying ingested steamed rice (Time _50%_) were compared by Wilcoxon rank sum test. A *p* value <0.05 was taken to indicate significant differences.

## Results

Effects of changing meal sequence (RF, FR and MR) on glucose excursion, and secretions of insulin and glucagon were assessed. In FR and MR, in comparison with RF, the rapid glucose elevation after rice ingestion was suppressed in type 2 diabetes and controls (Fig. 1). FR and MR suppressed glucose elevation from 30 to 90 min in type 2 diabetes, but elevated glucose levels at 240 min. Similar suppressive effects by FR and MR on glucose elevation were observed in controls. Likewise, FR and MR lowered both AUC_−15–240 min_-glucose (Fig. 1) and SD_−15–240 min_-glucose (type 2 diabetes, FR 1.94 ± 0.22 mmol/l, MR 1.68 ± 0.18 mmol/l, RF 2.77 ± 0.24 mmol/l; healthy, FR 0.95 ± 0.21 mmol/l, MR 0.83 ± 0.16 mmol/l, RF 1.18 ± 0.27 mmol/l) in type 2 diabetes and controls; the reduction in AUC_−15–240 min_-glucose and SD_−15–240 min_-glucose reached statistical significance in type 2 diabetes but not in controls, possibly due to the milder glucose elevation after rice ingestion. No significant differences in AUC_−15–240 min_-glucose and SD_−15–240 min_-glucose were observed between FR and MR in type 2 diabetes or controls. To clarify the mechanism of suppressed glucose elevation by FR and MR, the levels of insulin, C-peptide and glucagon were examined (Fig. 1). Insulin and C-peptide levels after rice ingestion as well as AUC_−15–240 min_-insulin and AUC_−15–240 min_-C-peptide were reduced by FR and MR in T2DM and controls, compared with RF. Reduction of AUC_−15–240 min_-insulin in type 2 diabetes and reduction of AUC_−15–240 min_-C-peptide in type 2 diabetes and controls reached statistical significance. Levels of glucagon and AUC_−15–240 min_-glucagon were elevated by FR and MR in type 2 diabetes and controls, when compared with RF. Elevation of AUC_−15–240 min_-glucagon by FR in type 2 diabetes and by MR in type 2 diabetes and controls reached statistical significance. No significant differences in AUC_−15–240 min_-insulin, AUC_−15–240 min_-C-peptide, or AUC_−15–240 min_-glucagon were observed between FR and MR in type 2 diabetes and controls. Similar results in type 2 diabetes were obtained in experiments comparing FR and RF (ESM Fig. 1), MR and rice before meat (RM) (ESM Fig. 3), and MR and FR (ESM Fig. 5).

To investigate involvement of incretin secretion in the effects of FR and MR on glucose levels, GLP-1 and GIP were measured (Fig. 2). Interestingly, levels of GIP and GLP-1 were elevated by FR and MR in type 2 diabetes and controls, when compared with RF. Elevation of AUC_−15–240 min_-GLP-1 and AUC_−15–240 min_-GIP by MR in type 2 diabetes and controls and elevation of AUC_−15–240 min_-GLP-1 by FR in type 2 diabetes reached statistical significance. Furthermore, GIP levels were more strongly elevated in MR than FR in both type 2 diabetes and controls. The difference in AUC_−15–240 min_-GIP between FR and MR in type 2 diabetes reached statistical significance. The difference in AUC_−15–240 min_-GLP-1 between FR and MR in controls, but not in type 2 diabetes, also reached statistical significance. Similar results for type 2 diabetes were obtained in comparison with FR and RF (ESM Fig. 2), MR and RM (ESM Fig. 4), and MR and FR (ESM Fig. 6).

To clarify the mechanism by which FR and MR affect glucose levels, the gastric emptying rate was measured using a ^13^C-acetate breath test. FR and MR significantly delayed gastric emptying rate and extended Time_50%_, the time required for 50% of ^13^C-labelled acetate to exit the stomach, in type 2 diabetes and controls (Fig. 3). Interestingly, Time_50%_ was well correlated with AUC_−15–120 min_-glucose and SD_−15–240 min_-glucose in type 2 diabetes and controls (Fig. 4), indicating that extension of the gastric emptying rate of rice plays a pivotal role in suppression of glucose elevation in FR and MR. Correlation of Time_50%_ with AUC_−15–120 min_-glucagon (type 2 diabetes, *r* = 0.399, *p* < 0.05; controls, *r* = 0.471, *p* < 0.05) and AUC_−15–120 min_-GLP-1 (type 2 diabetes, *r* = 0.437, *p* < 0.05; controls, *r* = 0.300, *p* = 0.107) suggests involvement of glucagon and/or GLP-1 in this process.

## Discussion

We find in this study that altered meal sequence can improve postprandial glucose regulation through delayed gastric emptying in type 2 diabetes and healthy controls. FR and MR enhanced glucagon and GLP-1, both of which may be involved in delayed gastric emptying. We also found that GIP secretion is affected by meal composition as well as meal sequence.

Postprandial glucose excursion is influenced by many factors including meal size and composition, gastric emptying and intestinal glucose absorption, as well as secretion and action of insulin and glucagon and the incretins. The gastric emptying rate accounts for ~35% of the variance in the initial rise of postprandial glucose levels in healthy and type 2 diabetes individuals [8, 9]. Delayed gastric emptying is well correlated with glucose excursions and glucose variability (Fig. 4). Remaining questions include how FR and MR slow the gastric emptying rate. Previous studies found that nutrients in ingested foods, including proteins and fats, influence differences in gastric emptying rate, possibly through modulation of GLP-1 [25, 26]. While the nutrients in fish and meat responsible for the modulation of gastric emptying remain unknown, the levels of glucagon and GLP-1 were well correlated with the gastric emptying rate, indicating involvement of glucagon and/or GLP-1 in the process. In addition, it is possible that cholecystokinin, which is secreted from the duodenum and jejunum in response to ingestion of fat and protein and delays gastric emptying [8, 9], might also be involved in this process, but the hormone was not measured in the present study. While the second meal phenomenon, in which suppression of NEFA after preloading low-carbohydrate and protein-rich snacks 1–2 h before meals associates with amelioration of postprandial glucose excursions, has been discussed in health and type 2 diabetes [27–29], the current study found no NEFA suppression by fish or meat preload, indicating that the current finding differs from the second meal phenomenon (DY, unpublished observations).

Preload of fish or meat before rice similarly enhanced GLP-1 secretion (Fig. 2, and ESM Figs 2, 4, 6). We initially supposed that fish oils such as EPA and DHA might selectively enhance GLP-1 secretion; such fatty acid-enhanced GLP-1 secretion occurs in cultured cells and experimental animals [16, 17]. However, the similarity of the effects of fish and meat on GLP-1 secretion indicates little involvement of fish oils. This is consistent with our observation that oral ingestion of EPA and preload of EPA before meal ingestion did not enhance GLP-1 secretion in humans (DY, unpublished observations). It was previously reported that preload of olive oil, whey proteins or glutamine before ingestion of carbohydrates enhanced GLP-1 secretion and delayed gastric emptying [6, 7, 15, 30].

On the other hand, preload of meat promoted GIP secretion but fish did not (Fig. 2). The fish and the meat meal used in the current study differed in fatty acid composition but had similar amino acid composition (ESM Table 1). It is known that saturated and monounsaturated fats can enhance GIP secretion in humans [31, 32]. Higher GIP promotes high fat diet-induced fat accumulation [13], suggesting that preload of meat before carbohydrates chronically could result in increased fat accumulation and increased insulin resistance. Our group previously reported that the HbA_1c_-lowering effects of DPP-4i were significantly correlated with estimated daily fish intake, and, to a lesser degree, with that of meat [33]. The chronic effects of fish and meat preload prior to carbohydrate on the therapeutic efficacy of DPP-4i in randomised, controlled clinical trials needs be examined in future.

Enhanced incretin secretion was not correlated with changes in the secretion of insulin or glucagon in the current study. Insulin and C-peptide levels were decreased by fish and meat preload in type 2 diabetes and healthy individuals, despite enhanced secretions of GIP and GLP-1 that should stimulate insulin secretion glucose-dependently. Previous studies on preloading whey proteins or glutamine showed increased insulin secretion concomitant with enhanced GLP-1 secretions [6, 15, 30]; however, the study on preloading olive oil showed no such enhancement [7]. Gastric emptying appears to be delayed more strongly by olive oil than by whey proteins [6, 7]. It is possible that stronger inhibition of gastric emptying limits the glucose elevation, thereby minimising the enhancement of glucose-dependent insulin secretion by incretins. A similar scenario has been noted between short- and long-acting GLP-1RA [14, 34]: short-acting GLP-1RA has stronger effects on gastric emptying but limited action on postprandial insulin secretion. Thus, enhanced glucagon secretion due to slower gastric emptying by fish and meat preload could result from stimulatory effects of amino acids on glucagon secretion at early time points and/or delayed inhibitory effects of glucose at later time points [35]. In addition, the effects of GLP-1 and GIP on glucagon secretion are glucose-dependent [36, 37], and may have limited effects on glucagon secretion.

Meal sequence has similar effects in type 2 diabetes and controls, which raises questions. First, levels of insulin and C-peptide immediately after ingestion of rice are much lower in type 2 diabetes compared with controls, while AUC_−15–240 min_-insulin in type 2 diabetes and AUC_−15–240 min_-C-peptide is comparable between type 2 diabetes and controls (Fig. 1). These findings are consistent with previous findings that insulin secretion immediately after ingestion of glucose or mixed meal is substantially impaired in Japanese patients with even short duration of the disease [38, 39]. Second, GLP-1 levels after rice ingestion and AUC_−15–240 min_-GLP-1 were much higher in type 2 diabetes than in controls. While it has been demonstrated that glucose tolerance does not affect GLP-1 secretion after ingestion of glucose or mixed meal [38, 40], the GLP-1 response in a particular meal sequence might differ between type 2 diabetes and controls. Further investigations are needed to characterise the GLP-1 responses during altered meal sequences. Moreover, glucagon levels after rice ingestion and AUC_−15–240 min_-glucagon were similar in type 2 diabetes and controls. Although hypersecretion of glucagon after ingestion of glucose or mixed meal is well known in type 2 diabetes [38], this might not be observed in this study because our participants had only mild type 2 diabetes.

The current study has limitations. The present experiment was intended to examine the effects of meal sequence in a single meal in a workable fashion, and was designed with a uniform 15 min interval between fish or meat and rice, which is similar to the interval used in previous studies (30 min) but potentially more practicable [6, 7]. Additional investigation is required to clarify optimal time intervals as well as amount, form and type of foods. Prospective, randomised clinical trials also are required to evaluate the chronic impact of altered meal sequences in type 2 diabetes management and prevention. In addition, the underlying mechanism of delayed gastric emptying by fish or meat preload remains largely unknown, although increased GLP-1 and glucagon levels are important in the process. The mechanisms by which altered meal sequence links to changes in the secretions of GLP-1 and GIP remain unclear, despite recent findings on the molecular regulation of incretin secretions [41]. Studies using cultured cells and experimental animals are in progress to identify possible dietary and pharmacological targets for management of postprandial glucose excursions. Although a ‘rice before meat’ arm might help to complete the current findings, it was not included to lighten the burden of the participants, and the results were expected from our preliminary studies (ESM Figs 1–6).

In conclusion, meal sequence is an important regulator of gastric emptying rate and postprandial glucose elevation through GLP-1 and glucagon secretions in individuals both with and without type 2 diabetes. Our current findings on meal sequence and composition with regard to the incretin secretions provide clues for medical nutrition therapy to better prevent and manage type 2 diabetes.

## Electronic supplementary material

ESM Fig. 1 (PDF 328 kb)

ESM Fig. 2 (PDF 273 kb)

ESM Fig. 3 (PDF 326 kb)

ESM Fig. 4 (PDF 272 kb)

ESM Fig. 5 (PDF 273 kb)

ESM Fig. 6 (PDF 245 kb)

ESM Table 1 (PDF 60 kb)

## Abbreviations

DHA: Docosahexanoic acid
DPP-4i: Dipeptidyl peptidase-4 inhibitors
EPA: Eicosapentaenoic acid
FR: Fish before rice
GIP: Glucose-dependent insulinotropic polypeptide
GLP-1: Glucagon-like peptide-1
GLP-1RA: Glucagon-like peptide-1 receptor agonists
MR: Meat before rice
RF: Rice before fish
RM: Rice before meat
