# Supplementary material for: Meal sequence and glucose excursion, gastric emptying and incretin secretion in type 2 diabetes: a randomised, controlled crossover, exploratory trial
Source: Diabetologia. 2015 Dec 24;59:453–61. doi: 10.1007/s00125-015-3841-z (PMC4742500; doi:10.1007/s00125-015-3841-z)
Supplement: Supplementary file 2 — (PDF 273 kb) [file 125_2015_3841_MOESM2_ESM.pdf]

ESM Fig. 2

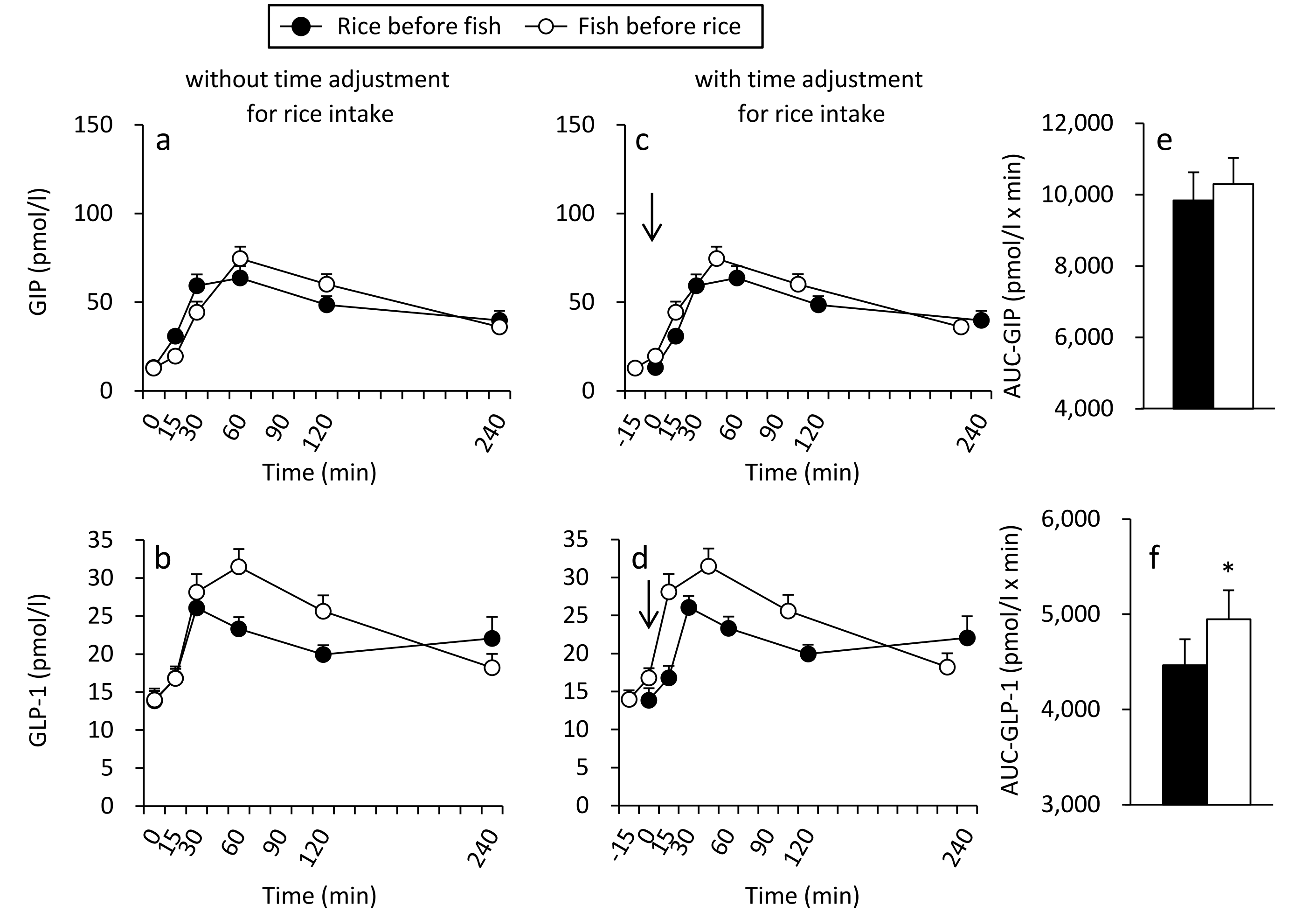

ESM Fig 2. Effects of meal sequence (rice before fish or fish before rice) on postprandial levels of glucose-dependent insulinotropic polypeptide (GIP) and glucagon-like peptide-1 (GLP-1) in patients with type 2 diabetes. Patients with type 2 diabetes [n=13; Age 57.6±4.3 year old; BMI 27.1±1.2 kg/m^2; Estimated duration of the disease 0.6±0.2 years; HbA_{1c} 6.9±0.6% (52.1±6.6mmol/mol)] were recruited and subjected to meal sequence tests on two separate mornings after overnight fasting. The patients received steamed rice (1004kJ) and boiled mackerel (920kJ) in two different meal sequences, rice before fish (RF) or fish before rice (FR), in a 2 way cross-over fashion. Unlike the experiment described in the main text, time for patients to receive 1st dish is defined as 0. Second dish was taken 15 min after the 1st dish. Time course curves are indicated for each measurement (RF, closed circles; and FR, open circles) (a-d). Area-under-the curves (AUC) for indicated measurements are shown (RF, closed bars; and FR, open bars) (e,f). p values for differences due to sequence (X), time (Y), and the interaction of sequence and time (Z) were calculated by mixed effects models as follows: (a) X0.675, Y0.000, and Z0.002; and (b) X0.013, Y0.000, and Z0.001. AUCs were analyzed by Wilcoxon's rank sum test, and * indicates p<0.05 for RF *versus* FR.
