# Supplementary material for: Meal sequence and glucose excursion, gastric emptying and incretin secretion in type 2 diabetes: a randomised, controlled crossover, exploratory trial
Source: Diabetologia. 2015 Dec 24;59:453–61. doi: 10.1007/s00125-015-3841-z (PMC4742500; doi:10.1007/s00125-015-3841-z)
Supplement: Supplementary file 3 — (PDF 326 kb) [file 125_2015_3841_MOESM3_ESM.pdf]

ESM Fig. 3

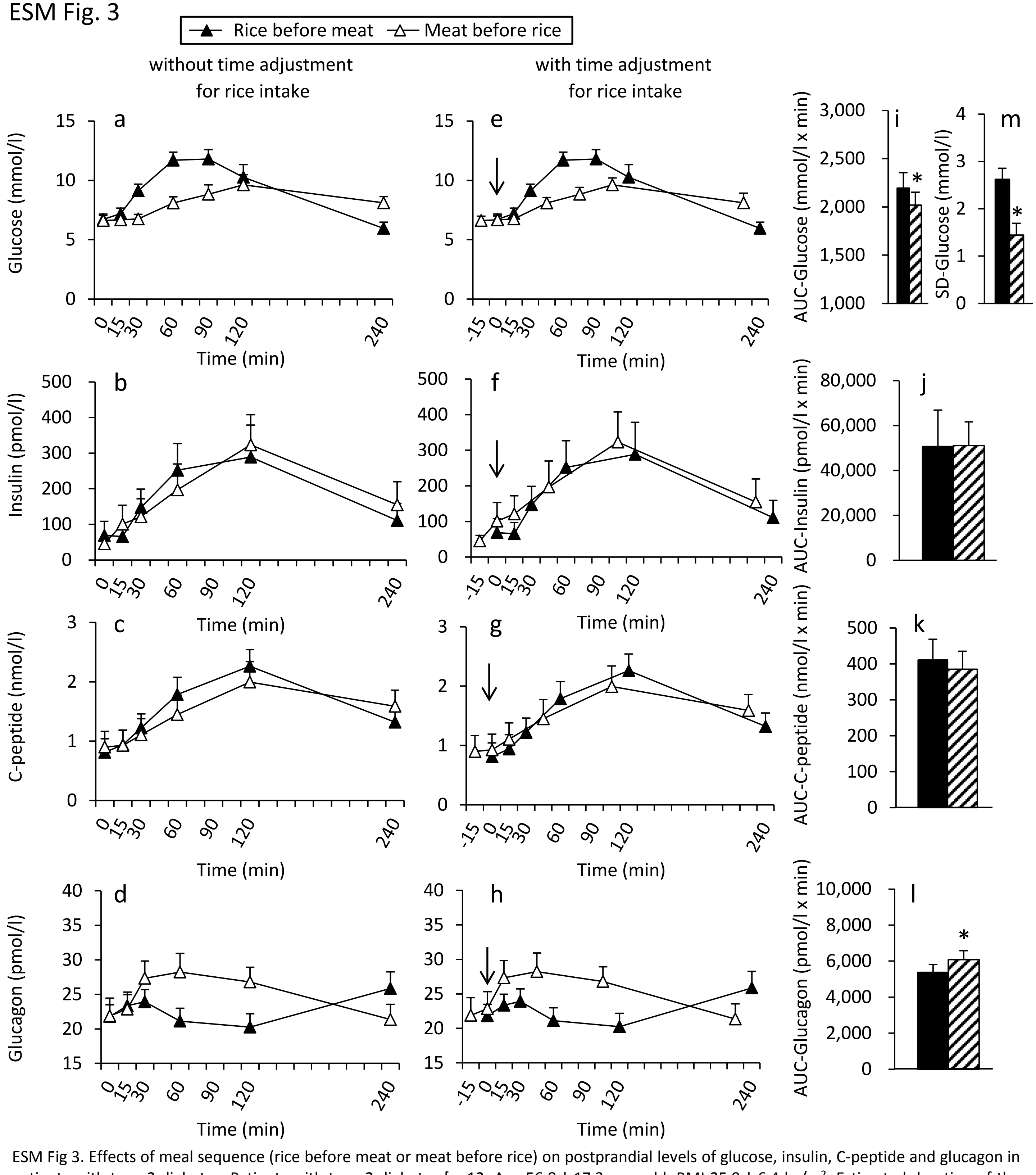

ESM Fig 3. Effects of meal sequence (rice before meat or meat before rice) on postprandial levels of glucose, insulin, C-peptide and glucagon in patients with type 2 diabetes. Patients with type 2 diabetes [n=12; Age 56.8±17.3 year old; BMI 25.9±6.4 kg/m^2; Estimated duration of the disease 4.5±4.3 years; HbA$_{1c}$ 6.8±1.1% (50.5±12.3mmol/mol)] were recruited and subjected to meal sequence tests on two separate mornings after overnight fasting. The patients received steamed rice (1004kJ) and grilled beef (920kJ) in two different meal sequences, rice before meat (RM) or meat before rice (MR), in a 2 way cross-over fashion. Unlike the experiment described in the main text, time for patients to receive 1st dish is defined as 0. Second dish was taken 15 min after the 1st dish. Time course curves are indicated for each measurement (RM, closed triangles; and MR, open triangles) (a-h). Area-under-the curves (AUC) for indicated measurements and standard deviations (SD) for glucose excursion are shown (RM, closed bars; and MR, hatched bars) (i-m). p values for differences due to sequence (X), time (Y), and the interaction of sequence and time (Z) were calculated by mixed effects models as follows: (a) X0.000, Y0.000, and Z0.000; (b) X0.000, Y0.953, and Z0.576; (c) X0.000, Y0.317, and Z0.065; and (d) X0.001, Y0.016, and Z0.000. AUCs and SDs were analyzed by Wilcoxon's rank sum test, and * indicates $p<0.05$ for RM *versus* MR.
