# Supplementary material for: Meal sequence and glucose excursion, gastric emptying and incretin secretion in type 2 diabetes: a randomised, controlled crossover, exploratory trial
Source: Diabetologia. 2015 Dec 24;59:453–61. doi: 10.1007/s00125-015-3841-z (PMC4742500; doi:10.1007/s00125-015-3841-z)
Supplement: Supplementary file 7 — (PDF 60 kb) [file 125_2015_3841_MOESM7_ESM.pdf]

ESM Table 1. Nutritional compositions of boiled mackerel, grilled beef and steamed rice used in the current study.

		Boiled mackerel	Grilled beef	Steamed rice
Energy (kJ)		920	920	1004
Weight (g)		100.0	79.1	150.0
Protein (g)		15.1	16.4	3.5
Fat (g)		17.7	17.1	0.6
Carbohydrate (g)		0.0	0.2	53.4
Salt (g)		0.7	0.6	0.2
Saturated fatty acids	Myristic acid (C14:0) (mg)	1100	309	N.D.
	Pentadecylic acid (15:0) (mg)	86	87	N.D.
	Palmitic acid (16:0) (mg)	2200	4827	N.D.
	Heptadecanoic Acid (17:0) (mg)	0	206	N.D.
	Stearic acid (18:0) (mg)	480	3640	N.D.
Monounsaturated fatty acids	Myristoleic acid (14:1) (mg)	0	67	N.D.
	Palmitoleic acid (16:1) (mg)	620	459	N.D.
	Oleic acid (18:1) (mg)	1700	7122	N.D.
	Eicosenoic acid (20:1) (mg)	370	69	N.D.
	Docosenoic acid (22:1) (mg)	95	0	N.D.
	Tetracosenoic acid (24:1) (mg)	140	0	N.D.
Polyunsaturated fatty acids	Linoleic acid (18:2, n-6) (mg)	280	206	N.D.
	α-Linolenic acid (18:3, n-3) (mg)	270	0	N.D.
	Arachidonic acid (20:4, n-6) (mg)	130	0	N.D.
	Eicosapentanoic acid (20:5, n-3) (mg)	5400	0	N.D.
	Docosapentanoic acid (22:6, n-3) (mg)	2200	0	N.D.
Amino acids	Arginine (g)	0.78	1.16	N.D.
	Lysine (g)	1.13	1.65	N.D.
	Histidine (g)	0.81	0.03	N.D.
	Phenylalanine (g)	0.51	0.74	N.D.
	Tyrosine (g)	0.45	0.65	N.D.
	Leucine (g)	0.99	1.49	N.D.
	Isoleucine (g)	0.58	0.83	N.D.
	Methionine (g)	0.36	0.48	N.D.
	Valine (g)	0.67	0.87	N.D.
	Alanine (g)	0.78	1.04	N.D.
	Glycine (g)	0.73	0.78	N.D.
	Proline (g)	0.50	0.68	N.D.
	Glutamate (g)	1.72	2.74	N.D.
	Serine (g)	0.52	0.71	N.D.
	Threonine (g)	0.59	0.84	N.D.
	Aspartate (g)	1.22	1.71	N.D.
	Tryptophan (g)	0.15	0.23	N.D.
	Cysteine (g)	0.13	0.22	N.D.

Nutritional compositions of boiled mackerel, grilled beef (sirloin) and steamed rice was conducted by Shokukanken Inc (Gunma, Japan). Energy contents were determined using modified Atwater factors (i.e., carbohydrate 16.736 kJ/g, protein 16.736 kJ/g, and fat 37.656 kJ/g). Protein contents were calculated by the Kjeldahl method using conversion factor 6.25. Fat contents were determined by the Soxhlet extraction method. Carbohydrate contents were calculated by subtracting contents of protein and fat as well as those of incinerated ashes measured by the direct flame incineration method and those of water measured by the atmospheric heating drying method from total weights. Salt contents were estimated by sodium contents determined by the atomic absorption spectrophotometry. Fatty acids were determined by gas chromatography-mass spectrometry after hydrolysis and subsequent methylation of fat extracts. Amino acids were determined by ion exchange column chromatography after oxidization with performic acid and subsequent hydrolysis with hydrochloric acid. N.D., not determined.
